# Salvianolic Acid B (Sal B) Protects Retinal Pigment Epithelial Cells from Oxidative Stress-Induced Cell Death by Activating Glutaredoxin 1 (Grx1)

**DOI:** 10.3390/ijms17111835

**Published:** 2016-11-03

**Authors:** Xiaobin Liu, Christy Xavier, Jamieson Jann, Hongli Wu

**Affiliations:** 1 Pharmaceutical Sciences, University of North Texas System College of Pharmacy, University of North Texas Health Science Center, Fort Worth, TX 76107, USA; xiaobin.liu@unthsc.edu (X.L.); Christy.Xavier@unthsc.edu (C.X.); jjann@uga.edu (J.J.); 2 North Texas Eye Research Institute, University of North Texas Health Science Center, Fort Worth, TX 76107, USA

**Keywords:** retinal pigment epithelial cells, salvianolic acid B, glutaredoxin 1, oxidative stress, protein glutathionylation

## Abstract

Protein glutathionylation, defined as the formation of protein mixed disulfides (PSSG) between cysteine residues and glutathione (GSH), can lead to cell death. Glutaredoxin 1 (Grx1) is a thiol repair enzyme which catalyzes the reduction of PSSG. Therefore, Grx1 exerts strong anti-apoptotic effects by improving the redox state, especially in times of oxidative stress. However, there is currently no compound that is identified as a Grx1 activator. In this study, we identified and characterized Salvianolic acid B (Sal B), a natural compound, as a Grx1 inducer, which potently protected retinal pigment epithelial (RPE) cells from oxidative injury. Our results showed that treatment with Sal B protected primary human RPE cells from H_2_O_2_-induced cell damage. Interestingly, we found Sal B pretreatment upregulated Grx1 expression in RPE cells in a time- and dose-dependent manner. Furthermore, NF-E2-related factor 2 (Nrf2), the key transcription factor that regulates the expression of Grx1, was activated in Sal B treated RPE cells. Further investigation showed that knockdown of *Grx1* by small interfering RNA (siRNA) significantly reduced the protective effects of Sal B. We conclude that Sal B protects RPE cells against H_2_O_2_-induced cell injury through Grx1 induction by activating Nrf2 pathway, thus preventing lethal accumulation of PSSG and reversing oxidative damage.

## 1. Introduction

The retinal pigment epithelium (RPE), lying between the photoreceptors and the choriocapillaris, is a monolayer of pigmented cells which plays essential roles in the retina, including maintaining the overlying photoreceptors, mediating the uptake of nutrients, ions, and water, phagocytizing the shed photoreceptor outer segment, and more. Several retinal degenerative diseases, including age-related macular degeneration (AMD), are closely related with RPE dysfunction [1]. Although the exact pathogenesis of AMD remains largely unknown, oxidative stress-induced RPE damage is believed to be one of the main causes [2,3]. Oxidative damage may lead to RPE morphological changes, including hyper- or hypopigmentation, vacuolation, disruption of tight junctions, and accumulation of extracellular deposition. Extensive degeneration of the RPE and the overlying photoreceptors, defined as geographic atrophy (GA), is the complication of dry AMD, and currently there is no available treatment [4]. As such, limiting oxidative stress in the RPE may represent an effective way to prevent, or even reverse, vision loss in patients with AMD. Understanding the function of antioxidant defense system in the RPE is, therefore, critical for developing new therapies for AMD.

Protein glutathionylation is defined as the formation of protein mixed disulfides (PSSG) between critical cysteine residues and glutathione (GSH) [5]. In GSH-abundant tissues, such as the retina, the most common type of protein oxidation is glutathionylation, which often affects the cysteines on the active sites of proteins and renders proteins dysfunctional [6]. High amounts of PSSG eventually lead to cell injury and death due to the inactivation of multiple important proteins and enzymes [7,8]. Therefore, reversing PSSG is vital to prevent cell damage and death. Specifically, the glutaredoxin family (Grxs) catalyzes the reduction of PSSG and, therefore, functions as powerful protein thiol oxidation repair enzymes [9,10,11]. In mammalian cells, Grxs exist in two subsets: Grx1 and Grx2. Grx1 is primarily localized to the cytoplasm, but has been implicated in the nucleus and inner mitochondrial membrane. Concentrated in the mitochondria ten times more than Grx1, Grx2 primarily exerts its protective effects in the mitochondrial matrix but has been associated with the nucleus, as well [12,13]. Grx1 and Grx2 have a significant Cys-X-X-Cys active site motif. The two cysteine residues act as redox sensors and allow for a monothiol mechanism using the *N*-terminal cysteine to reduce the reduced sulfhydryl groups of the cysteines of proteins. 

The distribution of Grx1 systems in the ocular tissues was firstly analyzed by Wu and Lou, and they found Grx1 expression and activity in most ocular tissues, including the cornea, iris, lens, ciliary body, and retina [14]. The same group also found that Grx1 protected lens epithelial cells from oxidative stress by reactivating the oxidatively-inactivated glyceraldehyde 3-phosphate dehydrogenase (G3PD) through dethiolation [15]. Grx1 is also critical for protecting human RPE cells against oxidative stress, and our previous study has shown that Grx1 overexpression protected RPE cells from H_2_O_2_-induced apoptosis by stimulating AKT phosphorylation through prevention of AKT glutathionylation [16]. Considering Grx1’s protective abilities in RPE cells, Grx1 could be a potential pharmacological target for retinal degenerative diseases, like AMD. Therefore, identifying Grx1 inducers may provide a novel strategy for AMD treatment. 

Danshen, the dried root of *Salvia miltiorrhiza*, has been recorded as a “superior grade” medicine in Shen-nung Pen-tsao Ching, the earliest materia medica extant in China [17]. It has been widely used in many Asian countries for the treatment of cardiovascular disorders and cerebrovascular diseases for thousands of years. This herb contains a large number of active natural compounds which have been reported to have significant free radical scavenging effects and protective effects on heart and brain injuries induced by ischemia-reperfusion [18]. Salvianolic acid B (Sal B) is the most abundant water-soluble phenol-rich antioxidant compound extracted from Danshen and, therefore, accounts for most of its therapeutic efficacy. Previous studies have shown that Sal B has strong antioxidant activities, as indicated by preventing lipid peroxidation, inhibiting malondialdehyde formation, and decreasing inflammatory factor-mediated membrane permeability in vascular endothelial cells [19]. Several studies have identified the therapeutic potential of Sal B as a free radical scavenger [20], a neuroprotectant [21], and a molecular chaperone inducer [22]. However, the protective activity of Sal B in primary cultured human RPE cells and its effects on Grx system has not yet been explored. Here, we demonstrated that Sal B protects RPE cells from H_2_O_2_-induced cell injury through up-regulating Grx1 to prevent the oxidation of critical sulfhydryl proteins.

## 2. Results

### 2.1. Salvianolic Acid B (Sal B) Prevents H_2_O_2_-Induced Cell Damage and Death in a Dose- and Time-Dependent Assay

**Figure 1:**
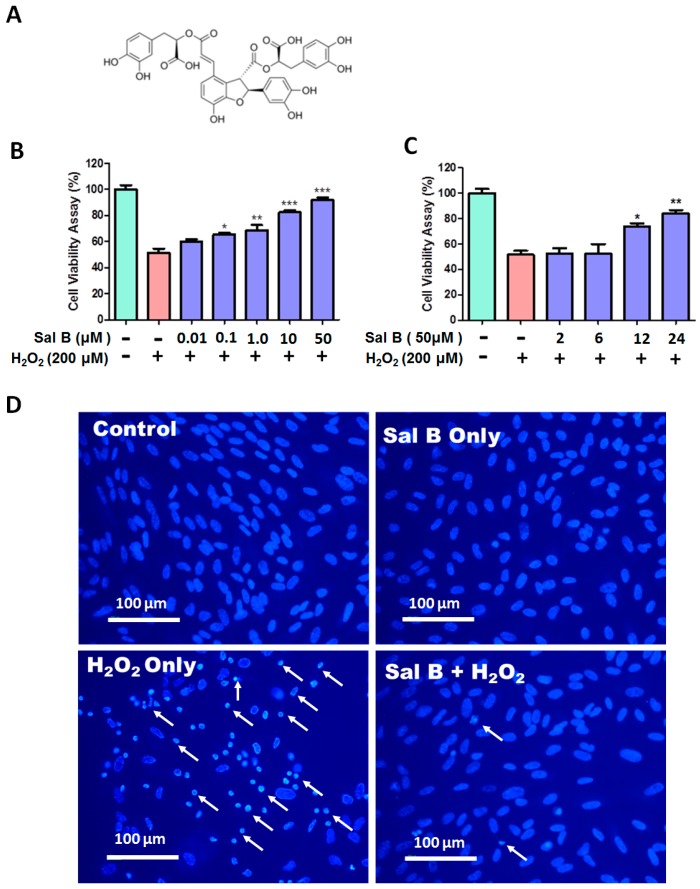
The protective effects of salvianolic acid B (Sal B) on H_2_O_2_-induced cytotoxicity in retinal pigment epithelial (RPE) cells. (**A**) The chemical structure of Sal B; (**B**) dose-dependent cytoprotective effects of Sal B. RPE cells were first pretreated with or without Sal B (0.01–50 µM) for 24 h and then incubated in 200 µM H_2_O_2_ for 6 h; (**C**) time-dependent cytoprotective effects of Sal B. RPE cells were pretreated with or without 50 µM Sal B for 2, 6, 12, and 24 h and then treated with 200 µM H_2_O_2_ for another 6 h. Cell viability was measured via a WST-8 assay and represented by mean ± SEM. * *p* < 0.05, ** *p* < 0.01, *** *p* < 0.001 compared with the H_2_O_2_ treated group (*n* = 8); and (**D**) cell apoptosis in Sal B treated cells. RPE cells were first pretreated with or without 50 µM Sal B for 24 h and then incubated in 200 µM H_2_O_2_ for 6 h. Cells were then subjected to Hoechst 33342 staining. Apoptotic cells are labeled with white arrows as having a nuclear shrinkage or strong fluorescence (*n* = 3).

The molecular structure of Sal B was shown in Figure 1A. As shown in Figure 1B, 200 μM H_2_O_2_ treatment was used because it caused 50% cell viability loss in RPE cells. With increasing doses of Sal B, RPE cell viability significantly increased. Sal B treatment of 50 μM provided the greatest protection, restoring more than 90% cell viability. Similarly, Sal B exerted its cytoprotective effects in a time-dependent manner as well (Figure 1C). The most significant cellular protective effects were seen at 12 and 24 h time points, with 75% ± 2.8% and 93% ± 3.1% cell viability, respectively.

### 2.2. Sal B Treatment Reduces Apoptosis in H_2_O_2_-Treated Retinal Pigment Epithelial (RPE) Cells

Hoechst staining was used to measure the anti-apoptotic effects of Sal B. Sal B treatment alone and control cells maintained uncondensed chromatin with dull blue fluorescence, demonstrating that cells were healthy. With H_2_O_2_ treatment, the nuclei of these cells were stained with very bright blue fluorescence. These nuclei have highly condensed chromatin, which showed up as crescents around the periphery of the nucleus (Figure 1D). This indicated that H_2_O_2_ treatment generated a high amount of cell apoptosis. However, with Sal B pretreatment, dull blue fluorescence and normal morphology were returned, indicating Sal B effectively protected RPE from oxidative stress-induced apoptosis.

**Figure 2:**
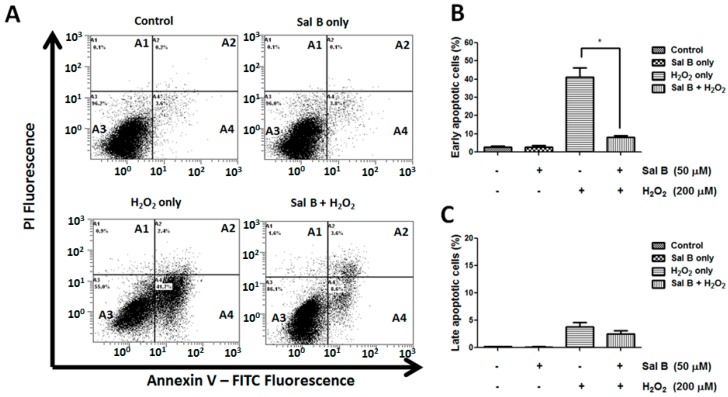
Sal B decreases apoptotic cell death in H_2_O_2_-treated RPE cells. (**A**) Flow cytometry of annexin V/propidium iodide (PI) double stained control, Sal B treatment alone, H_2_O_2_-treated only, and 50 μM Sal B and H_2_O_2_-treated RPE cells, showing live cells in quadrant A3, early apoptotic cells in quadrant A4, late apoptotic cells in quadrant A2, and necrosis in quadrant A1. Representative figures showing the populations of viable (annexin V^−^/PI^−^), early apoptotic (annexin V^+^/PI^−^), late apoptotic (annexin V^+^/PI^+^), and necrotic (annexin V^−^/PI^+^) cells. Bar graphs showing the quantification of early (**B**), and late apoptotic (**C**) cells. Data was presented as mean ± SEM of three independent experiments. * *p* < 0.05 compared with the H_2_O_2_-only group. FITC, fluorescein isothiocyanate.

Next, annexin V/PI double staining method was used to quantify apoptotic cells. The representative images for flow cytometry and the summarized data are presented in Figure 2. Cell apoptosis levels were equally low in both control and Sal B treatment alone cells. However, after H_2_O_2_ treatment, the rate of early apoptosis increased to 41.7% ± 4.9% but remained very low (4.8% ± 0.5%) in the Sal B pretreated group (*p* < 0.05) (Figure 2B). Furthermore, after exposure to H_2_O_2_, the number of late apoptotic cells slightly increased to 4.7% ± 1.8%, and pretreatment with Sal B reduced the percentage of late apoptosis to 2.8% ± 0.9% (Figure 2C). Taken together, this data strongly suggest that Sal B has anti-apoptotic properties in RPE cells.

### 2.3. Sal B Has Strong Reactive Oxygen Species (ROS) Scavenging Activity

**Figure 3:**
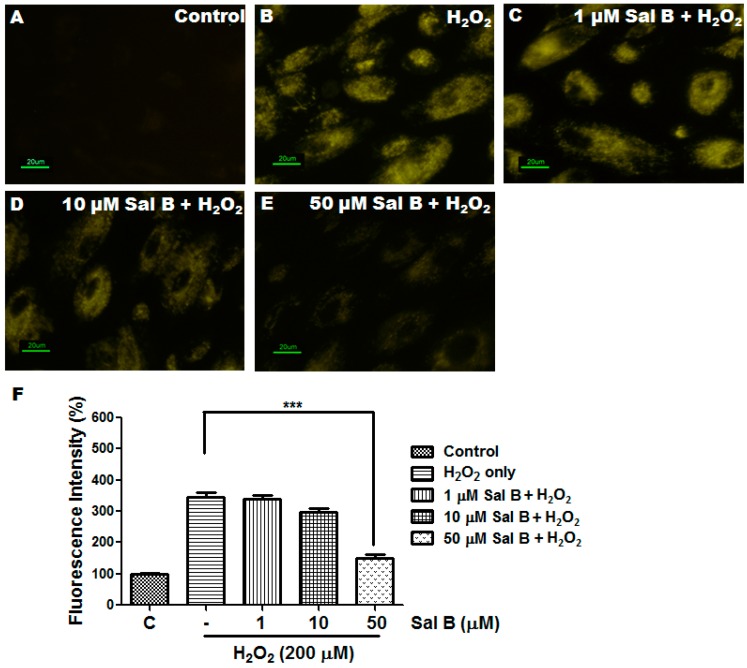
Sal B reduces reactive oxygen species (ROS) production in H_2_O_2_-treated RPE cells. RPE cells as a control with no treatment (**A**); and pretreatment without (**B**); or with 1 (**C**); 10 (**D**); and 50 μM (**E**) Sal B for 24 h, followed by 200 µM H_2_O_2_ treatment for 30 min. Fluorescence was detected using the probe CellROX orange reagent for all groups; and (**F**) fluorescence intensity quantified and represented as the mean ± SEM of three independent experiments, *** *p* < 0.001 compared with the H_2_O_2_-only group.

To measure reactive oxygen species (ROS) scavenging capabilities of Sal B, a CellROX orange reagent staining was performed to quantify the amount of ROS in Sal B pretreated cells. Figure 3 shows the fluorescence in live RPE cells by confocal microscopy 30 min after 200 μM H_2_O_2_ exposure. The higher the fluorescence intensity, the more ROS remains, and vice versa. As shown in Figure 3A, no fluorescence could be detected at the same time interval in the control cells where no H_2_O_2_ was added. In the absence of Sal B, H_2_O_2_ treatment significantly increased the fluorescence intensity of ROS (Figure 3B). Addition of Sal B gradually decreases the intracellular fluorescence intensity and almost completely suppresses it at a concentration of 50 μM (Figure 3C–E). Quantitative fluorescence intensities of CellROX Orange in the various groups are shown in Figure 3F.

### 2.4. Sal B Decreases Protein Glutathionylation in RPE Cells

**Figure 4:**
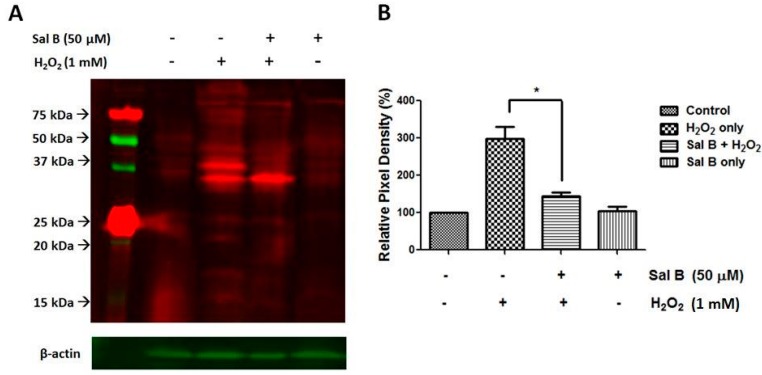
Sal B inhibits protein-glutathione mixed disulfide (PSSG) accumulation. (**A**) PSSG levels were detected by using Western blot. Cells were pretreated with or without 50 μM Sal B for 24 h followed by 1 mM H_2_O_2_ for 30 min. Total proteins were separated on a 12% SDS-gel under non-reducing condition (no β-mercaptoethanol) for Western blot analysis using an anti-PSSG antibody; PSSG bands were in red and actin bands were in green; (**B**) the relative pixel density of all the PSSG bands compared to β-actin. Data are mean ± SEM of three independent experiments, * *p* < 0.05 compared with the H_2_O_2_-only group (*n* = 3).

PSSG, the covalent modification of reactive protein cysteines with glutathione, is often a marker of protein oxidation, which can lead to cell damage and death. Therefore, a Western blot was conducted to see whether Sal B could reduce the amount of protein glutathionylation in RPE cells. As the protein glutathionylation process is relatively fast, we chose to treat the cells with a higher concentration of H_2_O_2_ (1 mM) for 30 min to compare the oxidation-induced PSSG formation in different experimental groups. As shown in Figure 4, in normal and Sal B treatment only cells, protein glutathionylation was present at normal amounts. With H_2_O_2_ treatment, protein glutathionylation was significantly elevated. However, 50 μM Sal B-pretreated RPE cells had much lower protein glutathionylation than with H_2_O_2_ treatment alone. This establishes Sal B as a capable compound that can prevent harmful protein glutathionylation.

### 2.5. Sal B Time- and Dose-Dependently Up-Regulates Glutaredoxin 1 (Grx1)

**Figure 5:**
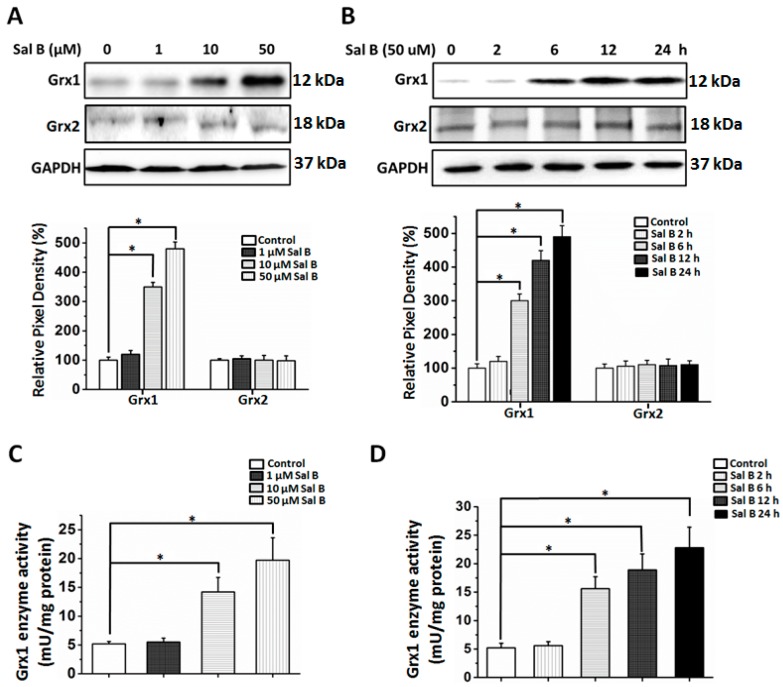
Sal B up-regulates Glutaredoxin 1 (Grx1) but not Grx2 in RPE cells. (**A**) Dose-dependent effects of Sal B on Grx1 and Grx2 expressions. RPE cells were treated with (1–50 μM) Sal B for 24 h. Control cells were treated identically but without addition of Sal B. Bottom panel: The relative pixel density of Grx1 and Grx2 over glyceraldehyde 3-phosphate dehydrogenase (GAPDH). * *p* < 0.05 compared with the control group (*n* = 3); (**B**) time-dependent effects of Sal B on Grx1 and Grx2 expression. RPE cells were treated with or without Sal B for 2, 6, 12, and 24 h. Bottom panel: The relative pixel density of Grx1 and Grx2 over GAPDH. * *p* < 0.05 compared with the control group (*n* = 3); (**C**) dose-dependent effects of Sal B on Grx1 enzyme activity. RPE cells were treated with (1–50 μM) Sal B for 24 h. Control cells were treated identically but without addition of Sal B. * *p* < 0.05 compared with the control group (*n* = 3); and (**D**) time-dependent effects of Sal B on Grx1 enzyme activity. * *p* < 0.05 compared with the control group (*n* = 3).

PSSGs are known to be the major substrate of Grx1. Sal B treatment markedly prevented H_2_O_2_-induced PSSG accumulation. This led us to explore whether Grx1 activation was associated with the cytoprotective effects of Sal B. In order to investigate the effects of Sal B on the expression of Grx1, RPE cells were treated with different concentrations of Sal B (1–50 μM) for 24 h. As shown in Figure 5A, Sal B dose-dependently upregulated Grx1. Sal B also increased Grx1 expression in a time-dependent manner. In RPE cells treated with 50 μM Sal B, the level of Grx1 increased significantly at 6 h after treatment and remained at a high expression level for 24 h (Figure 5B). Grx1 enzyme activity was also examined to confirm the Grx1-inductive effects of Sal B. As shown in Figure 5C,D, Sal B increased Grx1 enzyme activity in both time- and dose-dependent manners. In contrast, Grx2 expression was relatively unchanged by Sal B treatment in a time- and dose-dependent manner (Figure 5A,B), signifying that Sal B does not regulate Grx2 expression and may specifically induce Grx1 expression.

### 2.6. Sal B Up-Rregulates Grx1 through the NF-E2-Related Factor 2 (Nrf2) Transcriptional Pathway

**Figure 6:**
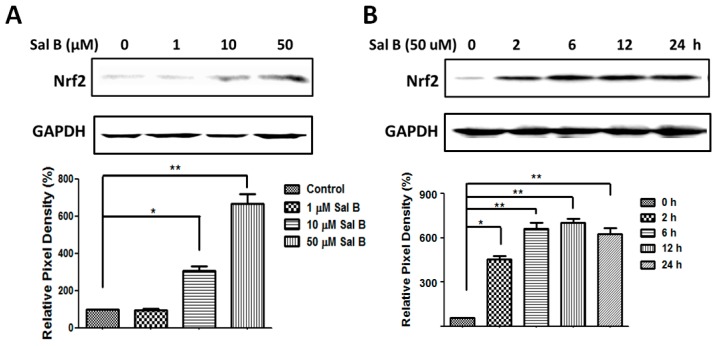
Sal B activates NF-E2-related factor 2 (Nrf2) pathway in RPE cells. (**A**) Dose-dependent effects of Sal B on Nrf2 expressions. RPE cells were treated with (1–50 μM) Sal B for 24 h. Control cells were treated identically but without addition of Sal B. Bottom panel: The relative pixel density of Nrf2 over GAPDH; (**B**) dose-dependent effects of Sal B on Nrf2 expression. RPE cells were treated with or without Sal B for 2, 6, 12, and 24 h. Bottom panel: The relative pixel density of Nrf2 over GAPDH. Data are mean ± SEM of three independent experiments, * *p* < 0.05, ** *p* < 0.01 compared with the control group.

NF-E2-related factor 2 (Nrf2), the major antioxidant transcriptional regulator, is responsible for inciting the transcription of multiple antioxidant enzymes, including Grx1. To prove that Sal B up-regulated Grx1 by Nrf2 activation, protein expression levels of Nrf2 were examined using Western blot. In Figure 6A, Nrf2 increased in a dose-dependent manner, with the highest Nrf2 expression seen at 50 μM Sal B treatment. Sal B exhibited similar effects in a time-dependent manner with a steady increase in Nrf2 expression. The level of Nrf2 increased as early as 2 h after Sal B treatment and maintained at a high expression level for 24 h (Figure 6B).

### 2.7. Sal B Protects Cells from Oxidative Stress-Induced Cell Injury by Promoting Grx1 Expression through Nrf2 Activation

**Figure 7:**
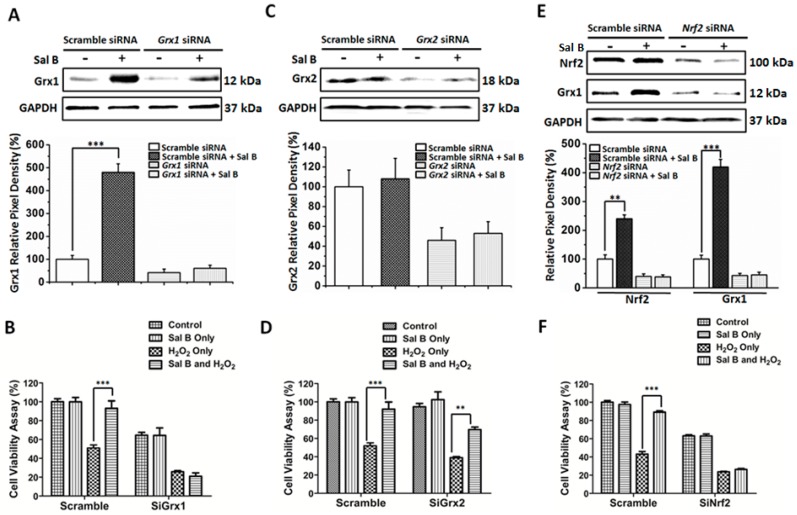
*Grx1* and *Nrf2* knockdown abolish the cytoprotective effects of Sal B. RPE cells were transfected with scramble or *Grx1* siRNA and then incubated with or without 50 μM Sal B for additional 24 h followed by H_2_O_2_ treatment for 6 h. (**A**) Western blot analysis of Grx1 protein levels. Total protein of each sample was subjected to Western blot with Grx1 and GAPDH antibodies. Bottom panel: the relative pixel density of Grx1 over GAPDH. *** *p* < 0.001 compared with the scramble siRNA control group (*n* = 3); (**B**) *Grx1* knockdown abolished the cytoprotective effects of Sal B. Cell viability in each group was measured using a WST-8 assay. Values are the mean ± SEM of three experiments, *n* = 6; *** *p* < 0.001 compared with the H_2_O_2_-treated group; (**C**) Western blot analysis of Grx2 protein levels. RPE cells were transfected with scramble or *Grx2* siRNA and then incubated with or without 50 μM Sal B for additional 24 h followed by H_2_O_2_ treatment for 6 h. Total protein of each sample was subjected to Western blot with Grx2 and GAPDH antibodies. Bottom panel: The relative pixel density of Grx2 over GAPDH. *** *p* < 0.001 compared with the scramble siRNA control group (*n* = 3); (**D**) *Grx2* knockdown did not change the cytoprotective effects of Sal B. Cell viability in each group was measured using a WST-8 assay. Values are the mean ± SEM of three experiments, *n* = 6; ** *p* < 0.01, *** *p* < 0.001 compared with H_2_O_2_-treated group; (**E**) *Nrf2* gene silencing inhibited Sal B-mediated Grx1 induction. RPE cells were transfected with scramble or *Nrf2* siRNA and then incubated with or without 50 μM Sal B for additional 24 h followed by H_2_O_2_ treatment for 6 h. Total protein of each sample was subjected to western blot with Nrf2, Grx1, and GAPDH antibodies. Bottom panel: the relative pixel density of Nrf2 and Grx1 over GAPDH. ** *p* < 0.01, *** *p* < 0.001 compared with the scramble siRNA control group (*n* = 3); and (**F**) *Nrf2* siRNA abolished the cytoprotective effects of Sal B. Cell viability in each group was measured using a WST-8 assay. Values are the mean ± SEM of three experiments (*n* = 6), *** *p* < 0.001 compared with H_2_O_2_-treated group.

The hypothesized mechanism of action of Sal B is that it has the capability to induce Grx1, leading to a better redox state in the cell. To prove this hypothesis, small interfering RNA (siRNA) was used to knockdown *Grx1* to see whether Sal B could maintain its antioxidant and protective effects. *Grx1* siRNA caused about a 50% reduction in Grx1 expression. However, with Sal B treatment, Grx1 expression was only slightly increased (Figure 7A). As seen in Figure 7B, 50 μM Sal B pretreated scramble siRNA RPE cells were more resistant to oxidative damage, having a cell viability of 80% after H_2_O_2_ treatment. However, with Grx1 inhibition, the protective effects that Sal B had on cell viability with H_2_O_2_ treatment is no longer noticeable, as Sal B pretreatment gives only 20% cell viability, just like H_2_O_2_ treatment alone. This loss of cytoprotection was not reciprocated when *Grx2* was inhibited using siRNA. *Grx2* siRNA resulted in an approximate 50% reduction in Grx2 expression and was unaffected by Sal B treatment (Figure 7C). When treated with Sal B, Grx2 inhibition still leads to partial recovery and cell survival from H_2_O_2_ treatment causing, approximately, a 70% cell viability (Figure 7D).

To assess whether Sal B-induced Grx1 activation is Nrf2-dependent, we knocked down *Nrf2* gene expression by RNAi. As shown in Figure 7E,F, *Nrf2* gene silencing inhibited the expression of Grx1 and abolished the cytoprotective effects of Sal B. Lower Nrf2 expression leads to a similar decrease in Grx1 expression (Figure 7E). Moreover, as seen in Figure 7F, *Nrf2* siRNA did not protect the cells from H_2_O_2_–induced oxidative injury even with Sal B pretreatment, with an unchanged 30% cell viability. Taken together, our results suggest that Sal B protected RPE cells against oxidative stress-induced injury through Grx1 induction via Nrf2 activation.

## 3. Discussion

Glutaredoxin (Grx) is an important member of the thiol-disulfide oxidoreductase family. Discovered nearly three decades ago, Grx1 has been discovered to have direct transferase, peroxidase, and other antioxidant capabilities [10,23]. More importantly, Grx1 functions to improve the redox state in the cell by increasing GSH levels and lowering protein glutathionylation levels. Although extensive studies have been done on the physiological role of Grx1 which all indicated Grx1 as a potential drug target, as far as we know, there is currently no drug that is identified as a Grx1 inducer. Our data, for the first time, clearly shows that Sal B can increase RPE survival and protection, and its protective mechanism entails stimulation of Grx1 by activating the Nrf2 pathway. The present study demonstrated that (1) Sal B protected primary human RPE cells from H_2_O_2_-induced cell death in a dose- and time-dependent manner; (2) Sal B reduced the total number of apoptotic and early apoptotic RPE cells treated by H_2_O_2_; (3) Sal B pretreatment restored the redox state in oxidatively-damaged cells, as indicated by less ROS production and protein glutathionylation; and (4) Sal B potently increased the expression of Grx1 via Nrf2 activation.

Sal B is known to be used as a treatment for the atherosclerotic vascular diseases, including coronary artery disease [24,25] and stroke [26,27]. Lin et al. [21] showed that Sal B protected neuron cells from amyloid plaque formation and stimulated differentiation, survival, and self-renewal for neurons. Moreover, in our previous study, we presented that Sal B protected human umbilical vein endothelial cells (HUVECs) from H_2_O_2_-induced cytotoxicity by activating endoplasmic reticulum (ER) chaperones, including glucose-regulated protein 78 (GRP 78) [22]. However, Sal B’s effects on RPE cells and possibly on degenerative eye diseases has not yet been explored. Our data, for the first time, verifies that Sal B is a potent antioxidant, lowering ROS and PSSG levels in H_2_O_2_-treated RPE cells. High PSSG levels are often an appropriate indicator of high oxidative stress and protein inactivation and damage [7,28]. Therefore, Sal B’s effectiveness in reducing ROS and PSSG levels makes it a promising drug for controlling cell damage and death. It also has anti-apoptotic abilities, as demonstrated by its time and dose-dependent cell viability increases and fewer total apoptotic cells than with H_2_O_2_ treatment alone.

When we further studied its mechanism of action, we found that Sal B has a strong Grx-inducing activity. Despite being overlooked as a secondary antioxidant system, recent studies have shown that Grx1 is a highly vital antioxidant enzyme that perpetuates cellular survival and protection. Recently, we were able to show that Grx1 prevents AKT *S*-glutathionylation and, therefore, protects RPE cells from oxidative damage [16]. Furthermore, Grx1 is a potent anti-apoptotic enzyme, known to directly regulate the expression of anti-apoptotic Bcl-2 and possibly the cleavage of caspase-3 [16]. Conversely, *Grx1* gene knockout increases cellular sensitivity to oxidative stress and inhibits cell proliferation in lens epithelial cells [15]. Grx1 can also catalyze reactions to reduce other critical small molecule antioxidants, like ascorbic acid to vitamin C [29]. Xing et al. [30] found that Grx1 can protect glyceraldehyde 3-phosphate dehydrogenase (G3PD), the key glycolytic enzyme for adenosine triphosphate (ATP) production, by removing the protein-GSH mixed disulfide at the active site. Considering Grx1’s prominent anti-oxidative properties, discovering a drug that can directly stimulate Grx1 in RPE cells might be a novel treatment for oxidative stress-related diseases, including AMD. As shown in Figure 5, following pretreatment with Sal B, there were specific increases of Grx1 expression in a time- and dose-dependent manner, indicating that Grx1-inducing activity of Sal B plays a critical role in its cytoprotective effects. To further prove this hypothesis, we used siRNA to down-regulate Grx1 expression in RPE cells and see if loss of Grx1 could attenuate the protective effects of Sal B. Our data showed that *Grx1* knockdown cells were more sensitive to oxidation than scramble siRNA-trasfected cells (Figure 7A,B). This result is consistent with our previous study which indicated Grx1 inhibition sensitizes human RPE cells to oxidative stress [16]. More importantly, we found Grx1 inhibition totally abolished the protective effects of Sal B, which suggests that the stimulatory effects of Sal B on Grx1 are critical for Sal B-mediated cytoprotection (Figure 7B). However, Grx2, its mitochondrial isoform, was not essential for Sal B’s protection and cell survival, as shown in Figure 5 and Figure 7C,D. This indicates that Sal B is highly specific to Grx1 stimulation, despite that Grx1 and Grx2 are considered to be part of the same oxidoreductase family.

To better understand how Sal B up-regulates Grx1 expression, we checked if Sal B could activate Nrf2 pathway. It was reported very recently that Grx1 expression is regulated by Nrf2 [31]. As a primary antioxidant transcriptional regulator, Nrf2 is often kept in an inactive bound state to Keap1 and targeted for ubiquitination every 20 min [32]. However, with electrophilic or oxidative stress, Nrf2 and Keap1 disassociates, allowing Nrf2 to translocate to the nucleus and coordinate the stimulation of a battery of antioxidant and survival genes including Grx1 [33]. In our study, we found when RPE cells were treated with Sal B, Nrf2 expression was markedly increased, and Nrf2 accumulation occurred in advance of Grx1 up-regulation (Figure 6). Our data are consistent with earlier reports demonstrating that Sal B can activate Nrf2 pathway in several other organ systems. Among them, the liver [34,35], lung [36], brain [37], kidney [38], and vascular endothelial cells [19] have all been shown to be protected by Sal B pretreatment via up-regulating Nrf2 and its downstream phase II detoxification genes. Our data and these reports all indicated that Sal B can act as a potent activator of Nrf2 both in vitro and in vivo. Furthermore, our data also suggested that Nrf2 signaling is responsible for Sal B’s Grx1-reviving effect, as *Nrf2* siRNA blocked Sal B-induced Grx1 up-regulation in RPE cells (Figure 7E). The restoration of cell viability after Sal B pretreatment was also significantly hindered with *Nrf2* siRNA groups, indicating that the Nrf2 pathway is vital for Sal B to mediate cellular protection and survival (Figure 7F). These data strongly suggested that the Nrf2 pathway is involved in Sal B-induced Grx1 up-regulation.

**Figure 8:**
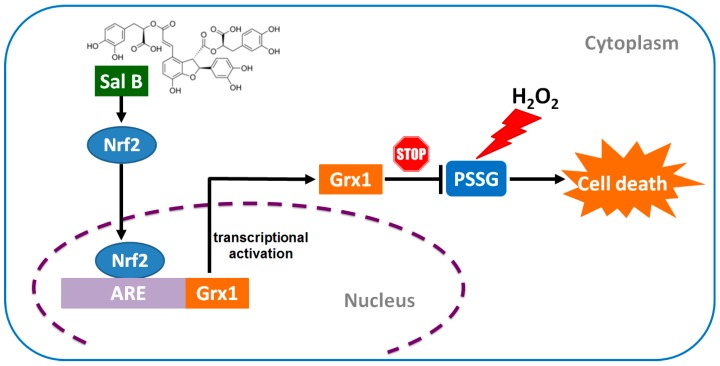
Sal B protected RPE cells by Grx1 induction though activating the Nrf2 pathway. Sal B in RPE cells acts upon Nrf2, causing Nrf2 expression to increase in the cell and translocate to the nucleus. Nrf2 binds to the antioxidant response element (ARE), causing the activation of Grx1 transcription. Grx1 up-regulation enhances cell survival due to an improved redox state and prevention of PSSG accumulation in RPE cells.

In summary, as shown in Figure 8, Sal B in RPE cells acts upon Nrf2, causing Nrf2 expression to increase in the cell and translocate to the nucleus. Nrf2’s binding on the ARE causes the induction of several cytoprotective enzymes especially Grx1. Grx1 up-regulation enhances cell survival and protection due to an improved redox state and prevention of PSSG accumulation in RPE cells. Sal B’s ability to stimulate Grx1 could be used to treat a variety of retinopathies and degenerative eye disorders that involve oxidative stress such as age-related macular degeneration. Therefore, considering its natural origin and distinguished success in promoting RPE cell survival, Sal B may have clinical potential for ophthalmic uses. Further investigation, including animal study, is required to better understand the retinal protective role of Sal B.

## 4. Materials and Methods

### 4.1. Materials

Salvianolic acid B was purchased from Sigma-Aldrich (49724, St. Louis, MO, USA). Dulbecco’s modified Eagle’s medium (DMEM; D5546), fetal bovine serum (FBS; F2442), penicillin/streptomycin (P4333), and 0.25% trypsin (T4049) and other cell culture reagents were purchased from Sigma-Aldrich (St. Louis, MO, USA). Hydrogen peroxide (216763), 2-hydroxyethyl disulfide (HEDS; 380474), glutathione (GSH; 1294820), glutathione reductase (GR; 10105678001), β-nicotinamide adenine dinucleotide phosphate-reduced tetra(cyclohexylammonium) salt (NADPH; N5130), and β-mercaptoethanol (M6250) were obtained from Sigma-Aldrich (St. Louis, MO, USA). Primary antibodies including anti-Grx1 (Abcam, Cambridge, UK, ab45953), anti-Grx2 (Abcam, ab191292), anti-PSSG (Virogen, London, UK, 101-A-100), anti-Nrf2 (Cell Signaling, Beverly, MA, USA, #12721), anti-GAPDH (Santa Cruz, CA, USA, sc-32233) antibodies, and horseradish peroxidase-conjugated secondary antibodies (Santa Cruz, sc2061, sc2060, sc2030) were purchased from Santa Cruz Biotechnology (Santa Cruz, CA, USA).

### 4.2. Human Retinal Pigment Epithelial (RPE) Cell Culture

Human fetal RPE cells were purchased at passage one from ScienCell™ Research Laboratories (6540; Carlsbad, CA, USA), and all experiments were performed with cells between passages two to five. The cells were maintained in DMEM supplemented with 10% FBS, 100 µg/mL streptomycin, and 100 U/mL of penicillin. Cell cultures were maintained at 37 °C in a humid atmosphere incubator with 5% CO_2_ and 95% air. The medium was changed every 3–4 days. To passage the cells, we subcultured RPE cells at a 1:4 dilution using 0.25% trypsin-ethylenediaminetetraacetic acid (EDTA) solution and cells usually reached confluence after about four days. Since the function of RPE is determined by cell–cell contact at confluence, in our study, only fully-confluent monolayer cells which exhibit hexagonal RPE morphology were used for the following experiments. To study the effects of H_2_O_2_ on apoptosis in RPE cells, cells were subjected to gradual serum deprivation. This involved culturing RPE cells in DMEM with 2% FBS and then placing the cells in serum-free medium for 30 min prior to six hours of 200 µM H_2_O_2_ treatment.

### 4.3. Cell Viability Assay

Cell viability was measured by a colorimetric cell viability kit (PK-CA705-CK04; Promokine, Heidelberg, Germany) with the tetrazolium salt WST-8 (2-(2-methoxy-4-nitrophenyl)-3-(4-nitrophenyl)-5-(2,4-disulfophenyl)-2*H*-tetrazolium, monosodium salt), which can be bioreduced to a water-soluble orange formazan dye by dehydrogenases present in the viable cells. The amount of formazan produced is directly proportional to the number of living cells. Cells were incubated with or without Sal B (0.1–50 μM) for 24 h and then treated with 200 µM H_2_O_2_ for 6 h. After treatment, 10 µL of WST-8 solution was added to each well of the culture plate and incubated for 2 h in the incubator. The absorption was evaluated at 450 nm using a microplate reader (BioTek, Winooski, VT, USA).

### 4.4. Hoechst 33342 Fluorescent Staining

RPE cells were incubated with or without 50 µM Sal B for 24 h and then treated with 200 µM of H_2_O_2_ for 6 h. Before fixing the cells with 4% paraformaldehyde for 15 min, ice-cold PBS was used to wash the cells. Cells were washed once more with PBS after 4% paraformaldehyde exposure. 0.1 µg/mL Hoechst 33342 (H3570, Invitrogen, Grand Island, NY, USA) was used to stain the fixed RPE cells for 10 min, and subsequently, the cells were rinsed with PBS. The resulting stained images were taken using a fluorescence microscope (Olympus, Center Valley, PA, USA).

### 4.5. Flow Cytometry Analysis of Cell Apoptosis

RPE cells were treated with or without 50 μM Sal B for 24 h followed by 200 μM H_2_O_2_ treatment for 6 h. After that, the cells were trypsinized and stained with annexin V and PI using annexin V/PI apoptosis Kit (V13242, Invitrogen, Grand Island, NY, USA) according to the manufacturer’s protocol. The stained cells were then analyzed by flow cytometer (FC500, Beckman Coulter, Indianapolis, IN, USA) to differentiate among viable (annexin V^−^/PI^−^), early apoptotic (annexin V^+^/PI^−^), late apoptotic (annexin V^+^/PI^+^) cells, and necrotic cells (annexin V^−^/PI^+^).

### 4.6. ROS Detection

Intracellular ROS levels were determined using the fluorescent probe CellROX orange reagent (10443; Life Technologies, Carlsbad, CA, USA). After pretreatment with 1–50 μM Sal B for 24 h, RPE cells were then exposed to 200 μM H_2_O_2_ for another 30 min. After the treatment, cells were loaded with 5 mM CellROX orange reagent for 30 min at 37 °C and washed with PBS. After treatment, the cells were loaded with 5 mM CellROX Orange Reagent for 30 min at 37 °C, washed with PBS and immediately imaged on a fluorescence microscope (Olympus, Center Valley, PA, USA). The cellular fluorescence was quantified using ImageJ software (National Institutes of Health, Bethesda, MD, USA), after background subtraction for each image.

### 4.7. Protein Glutathionylation Detection

RPE cells were pretreated with or without 50 μM Sal B for 24 h and then incubated with 1 mM H_2_O_2_ for 30 min. Cells were harvested by using radioimmunoprecipitation assay (RIPA) buffer (R0278; Sigma-Aldrich; St. Louis, MO, USA). Equal amounts of protein (80 μg) in each group were loaded onto a sodium dodecyl sulfate polyacrylamide gel electrophoresis (SDS-PAGE) under non-reducing (no β-mercaptoethanol) conditions, immunoblotted, and probed with anti-PSSG antibody.

### 4.8. Western Blot Analysis

Protein concentration was determined with a BCA assay kit (23225; Thermo Scientific, Rockford, IL, USA). Equal amounts of protein were boiled in Laemmli buffer (Bio-Rad, Bio-Rad, Richmond, CA, USA) and loaded onto 12% SDS-PAGE gel and transferred to a 0.2 µm polyvinylidene difluoride membrane (GE Healthcare, Boulder, CO, USA). The membranes were incubated with appropriate primary antibodies overnight at 4 °C and were then incubated with the appropriate secondary antibodies for 1 h. Detection was performed using the ECL Western blotting detection system (Thermo Scientific, Rockford, IL, USA). The immunoblot was analyzed with a Bio-Rad imaging system (Versadoc 5000 MP Imaging System, Bio-Rad, Richmond, CA, USA). 

### 4.9. Enzyme Activity Assays

Grx1 was assayed using HEDS as the substrate according to the method described in Raghavachari and Lou [12]. In brief, the cell lysate was mixed with potassium phosphate buffer (200 mM, pH 7.5) containing 0.5 mM GSH, 0.4 U/mL GR, 0.2 mM NADPH, and 2 mM hydroxyethyl disulfide (HED). The reaction was carried out at 30 °C, and the decrease in absorbance at 340 nm was monitored for 5 min and used to determine the Grx1 activity.

### 4.10. siRNA-Mediated Knockdown of Grx1, Grx2, and Nrf2 and Cell Viability Testing

Scramble, *Grx1*, *Grx2* and *Nrf2* siRNA used in this study were chemically synthesized by Santa Cruz Biotechnology (Santa Cruz, CA, USA). SiRNA duplexes (scrambled siRNA, sc-37007; *Grx1* siRNA, sc-72089; *Grx2* siRNA, sc-72090; *Nrf2* siRNA, sc-37030) were transfected into primary human RPE cell with siRNA transfection reagent (sc-29528; Santa Cruz, CA, USA) according to the manufacturer’s instructions. 

### 4.11. Statistics

All experiments were performed, at least, in triplicate in order to obtain statistically relevant data. The specific number of experimental samples used for each data set can be found in the figure legends. The Mann-Whitney *U* test allowed for accurate statistical analysis when comparing two groups. When comparing three or more groups, statistical analysis was done using the Kruskal-Wallis test followed by Dunn’s test as a post hoc test. All statistical analyses were conducted using Prism software (GraphPad, La Jolla, CA, USA). All of our numerical data are expressed as mean ± SEM in our figures and figure legends, and statistically significant differences were noted if *p* < 0.05.
